# Adsorption and Dissociation of 2-Chlorophenols on the 2D ZnO Monolayer Decorated with Al Atoms: A DFT Study

**DOI:** 10.3390/ma18040813

**Published:** 2025-02-13

**Authors:** Zhengjun Zong, Changqing Wang, Miaomiao Zhao, Weiguang Chen, Yu Jia

**Affiliations:** 1 Department of Mathematics and Physics, Luoyang Institute of Science and Technology, Luoyang 471023, China; 2 School of Physics and Electronic Engineering, Zhengzhou Normal University, Zhengzhou 450044, China; 3 Key Laboratory for Special Functional Materials of Ministry of Education, School of Physics and Electronics, Henan University, Kaifeng 475001, China

**Keywords:** adsorption, first-principles, dissociation, 2-chlorophenol, two-dimensional ZnO

## Abstract

The stable adsorption configurations, electronic structures, and dissociation properties of 2-chlorophenol on pristine and Al-decorated ZnO monolayer are investigated using density functional theory (DFT). Our results indicate that the interaction between 2-chlorophenol and pristine ZnO monolayer is weak, while Al-modified ZnO monolayer can significantly enhance the adsorption of 2-chlorophenol. Therefore, compared to the pristine ZnO monolayer, the ZnO monolayer modified with Al is more sensitive to 2-chlorophenol molecules. Moreover, both pristine ZnO and Al decorated ZnO monolayers exhibit lower barriers for the dissociation of 2-chlorophenol molecules. These results provide a deeper understanding of the adsorption and dissociation performance of the ZnO monolayer for 2-chlorophenol molecules, which will contribute to the further application of ZnO in the fields of catalysts and gas sensing.

## 1. Introduction

2-Chlorophenol is a highly toxic organic pollutant that serves as a precursor for catalyzing the formation of polychlorinated dibenzo-p-dioxins and polychlorinated dibenzofurans (PCDD/Fs) [1,2,3,4,5,6,7,8]. The adsorption and dissociation of 2-chlorophenol on the surface of metal oxides to form chlorophenoxy radical is a key step in the conversion of 2-chlorophenol to PCDD/Fs [9,10,11,12,13,14,15]. Chlorophenoxy radical, as an environmentally persistent free radical (EPFR), is a widely present pollutant in the environment. The removal or degradation of 2-chlorophenol and their environmental persistent free radicals is crucial for the governance and control of environmental pollution [7,13,14,15,16,17]. Therefore, studying the adsorption and dissociation process of 2-chlorophenol on the surface of metal oxides is crucial for understanding the formation mechanism of chlorophenoxy radicals.

Since the theoretical prediction that two-dimensional hexagonal honeycomb ZnO monolayers can exist stably [18], researchers have successfully prepared two-dimensional ZnO monolayers through various experimental methods [19,20,21,22,23]. In addition, it was observed that two-dimensional ZnO monolayers exhibit some excellent mechanical [24], optical [25,26], and catalytic [27,28,29] properties. Modifying the surface of two-dimensional materials with metal atoms to improve their catalytic performance is a commonly used experimental strategy [30,31,32,33]. The magnetic, electronic, and optical properties of two-dimensional ZnO monolayer can be adjusted by doping or adsorption of transition metal atoms [34,35,36,37,38]. Especially, metal atom modification can improve the gas sensitivity and catalytic performance of two-dimensional ZnO monolayers. For instance, by modifying with transition metal atoms, the adsorption and gas-sensing properties of the pristine ZnO monolayer for toxic small molecules (NO, CO, NH_3_) are significantly enhanced [39,40,41]. For aromatic molecules, such as benzene (C_6_H_6_) and toluene (C_7_H_8_), metal-decorated ZnO monolayers are the promising sensors with high selectivity and sensitivity [42]. More interestingly, doping with Al metal atoms can enhance the catalytic activity of ZnO materials toward CO oxidation [43] and improve significantly their gas sensitivity to CO [44].

In recent decades, the adsorption and dissociation mechanisms of organic toxic pollutant molecules (such as chlorophenols) on solid catalyst surfaces have been a hot topic in environmental pollution science research. The adsorption of 2-chlorophenol on Cu (100) [45] and Cu (111) [46] surfaces was studied using DFT. It was found that the binding of 2-chlorophenol molecules to these surfaces is very weak. Similarly, the dissociation mechanism of a single 2-chlorophenol molecule adsorbed on the surfaces of Cu_2_O to form a stable 2-chlorophenoxy, which is an environmentally persistent free radical, has also been investigated [9,10]. Thermal transformations of 2-chlorophenol on the surface of ZnO powder catalyst were investigated by Fourier-transform infrared spectroscopy (FTIR), X-ray photoelectron spectroscopy (XPS), and density functional theory (DFT) [11]. It was found that the dissociation barrier of C-Cl bond was significantly higher than that of O-H bond. Recently, adsorption of acetone onto the pristine and Al-doped ZnO nanotubes was explored by dispersion-corrected DFT [47]. It was shown that Al doping can significantly enhance the adsorption strength of ZnO nanotubes for acetone.

In the present study, we chose pristine and Al-modified two-dimensional honeycomb ZnO monolayers to investigate their adsorption and dissociation mechanisms toward 2-chlorophenol by first-principles calculations. Our investigation can help understand the formation mechanism of catalyzing polychlorinated dibenzo-p-dioxins and polychlorinated dibenzofurans (PCDD/Fs) and also contribute to the understanding of the gas-sensing properties of two-dimensional ZnO on 2-chlorophenol molecules and their environmental persistent free radicals (EPFRs).

## 2. Calculation Methods and Models

Based on DFT in the generalized gradient approximation (GGA) method, the exchange correlation energy was calculated using the Perdew–Burke–Ernzerhof (PBE) functional [48]. The pseudopotentials containing 12, 6, 4, 1, 7, 3, and valence electrons for the Zn (4s^2^ 3d^10^), O (2s^2^ 2p^4^), C (2s^2^ 2p^2^), H (1s^1^), Cl (3s^2^ 3p^5^), and Al (3s^2^ 3p^1^) ions were used, respectively. All calculations in the present study were implemented in the Vienna ab initio simulation package (VASP) [49,50]. The van der Waals (vdW) interaction with D3 Grimme correction (DFT-D3) [51] was used to better describe the long-range vdW interaction between ZnO monolayers and 2-chlorophenol molecules. The convergence standard for energy was set to 10^−5^ eV, and during the structural optimization process, the maximum force on each atom was less than 0.02 eV/Å. The cutoff energy of the plane wave basis set was taken as 520 eV. 5 × 5 × 1 Monkhorst-Pack mesh k-points were utilized in the DFT simulations. We employed the DFT+U method [52] to take into account the orbital-dependent Coulomb repulsion interaction between 3D electrons in the Zn atoms. As mentioned in reference [40], the Hubbard U value for Zn was set to 5 eV. We also examined the effect of different U (3–10 eV) values on the adsorption energy of 2-chlorophenol molecules on 2D ZnO surfaces. It was found that different U values have an impact on the adsorption energy within 0.01 eV. However, the energy difference between different adsorption positions of 2-chlorophenol molecules on the 2D ZnO surface remained unchanged. To avoid interlayer interactions caused by periodic boundary conditions, a 20 Å vacuum layer was used between two ZnO monolayers. Throughout the entire calculation process, crystal structure optimization was carried out using the conjugate gradient (CG) algorithm.

**Figure 1:**
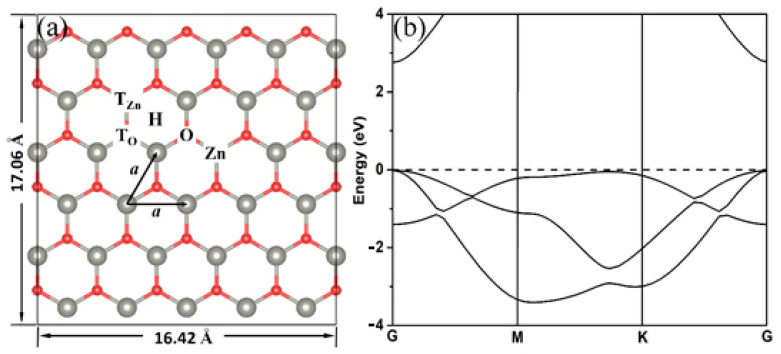
Schematic models (**a**) and the band structures (**b**) of 2D ZnO. Red and gray spheres represent O and Zn atoms, respectively. The Fermi energy level is set to zero.

A $5\times 3\sqrt{3}$ supercell containing 30 Zn atoms and 30 O atoms was constructed to simulate the 2D ZnO surface (Figure 1a). The lattice constant of 2D ZnO we calculated is *a* = 3.284 Å, which is in good agreement with the experimental value, 3.303 Å [19], and the theoretical value, 3.283 Å [25]. Therefore, the 2D ZnO surface is a 16.42 Å × 17.06 Å plane, ensuring that there is no interaction between the adjacent adsorbed 2-chlorophenol molecules. In addition, to verify the rationality of the parameters we selected, we calculated the band structure of 2D ZnO (as shown in Figure 1b), and the calculated bandgap was 2.77 eV, which is very consistent with the previous calculation results [24,25,26,27,28,29].

In order to better evaluate the relative stability of the considered adsorption system, the adsorption energy was calculated as follows:

(1) $$E_{ad}=E_{sub/\mathrm{adsorbate}}-E_{sub}-E_{\mathrm{adsorbate}}$$

where $E_{sub/\mathrm{adsorbate}}$, $E_{sub}$ and $E_{\mathrm{adsorbate}}$ are the total energy of adsorbate adsorbed on the substrate, the energy of the isolated substrate, and the energy of the isolated adsorbate, respectively. Negative adsorption energy indicates that the adsorption process of gas molecules on the substrate is exothermic, and a larger negative value indicates stronger binding between gas molecules and the substrate. The climbing image nudged elastic band method [53] (CI-NEB) was used to find the minimum-energy path (MEP). We inserted four images between two stable states, with a spring constant of −5.0 eV/Å between adjacent images. The electronic charge density difference $\Delta \rho =\rho_{sub+ad}-\rho_{sub}-\rho_{ad}$. $\rho_{sub+ad}$, $\rho_{sub}$, $\rho_{ad}$ represents the electronic charge density of the adsorption system, the substrate, and the adsorbate, respectively. It can reflect the electronic charge transfer at the interface.

## 3. Results and Discussion

### 3.1. Adsorption of 2-Chlorophenol on Pristine ZnO Monolayers

In order to find a stable adsorption structure of 2-chlorophenol on the 2D ZnO monolayer, two adsorption configurations, vertical and parallel to the ZnO monolayer, were considered. As shown in Figure 1, for each configuration, we considered three different adsorption positions: T_O_, in which the O atom of 2-chlorophenol is located at the top of the O atom in the 2D ZnO monolayer; T_Zn_, in which the O atom of 2-chlorophenol is located at the top of the Zn atom in the 2D ZnO monolayer; and H position, in which the O atom of 2-chlorophenyl is located at the hollow region of the 2D ZnO monolayer. Geometric parameters of the optimized 2-chlorophenol molecule adsorbed at different positions on a 2D ZnO monolayer are listed in Table 1. From Table 1, it can be seen that the adsorption energy of 2-chlorophenol molecules adsorbed parallel to the surface of ZnO is usually lower than that of vertical adsorption, indicating that parallel adsorption is more stable than vertical adsorption. In addition, the adsorption of 2-chlorophenol molecules on the top position of Zn is relatively stable. From the most stable adsorption configuration, 2-chlorophenol molecules are adsorbed parallel to the surface of ZnO (T_Zn_), with an adsorption energy of only −0.68 eV, an adsorption height of 2.56 Å, and a charge transfer of only 0.063 e. The adsorption of 2-chlorophenol molecules on the 2D ZnO monolayer is a weak adsorption. The interaction between 2-chlorophenol molecules and clean Cu_2_O [9,10] or Cu [45,46] surfaces also exhibits this weak adsorption.

**Figure 2:**
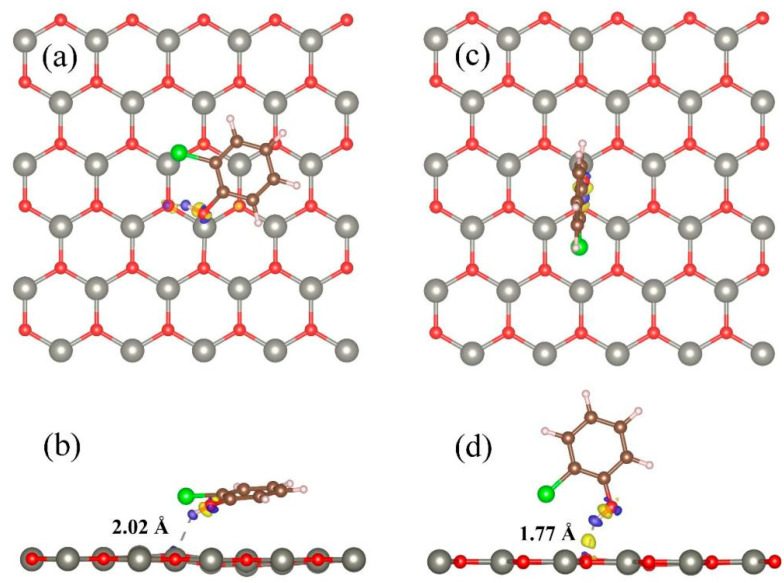
Charge density differences of optimized 2-chlorophenol molecule relatively stable parallel (**a**,**b**) and vertical (**c**,**d**) adsorption on the pristine ZnO monolayer. (**a**,**c**) TOP view (**b**,**d**) Side view. Yellow and blue correspond to charge accumulation and depletion, respectively. The isosurface value is 0.005 e/Å^3^. Red, gray, brown, green, and white spheres represent O, Zn, C, Cl, and H atoms, respectively.

The optimized relatively stable parallel and vertical adsorption configurations and corresponding charge density differences are shown in Figure 2. From Figure 2, it can be seen that when 2-chlorophenol molecules adsorb onto the surface of ZnO, only H atoms have weak hydrogen bonds with the O atoms in the surface of ZnO. The corresponding O-H bond lengths for parallel and vertical adsorption are 2.02 Å and 1.77 Å, respectively. This once again confirms that the adsorption of 2-chlorophenol molecules on the 2D ZnO monolayer is a weak adsorption.

**Figure 3:**
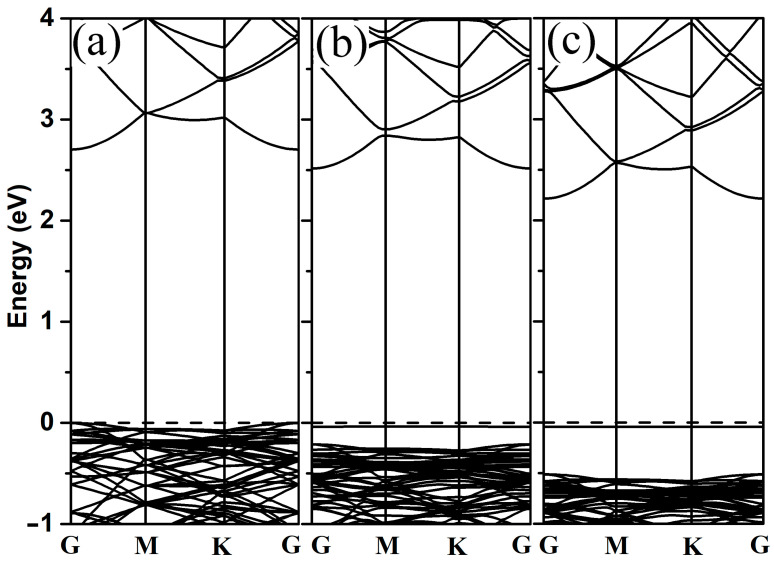
Band structures of pristine 2D ZnO (**a**), optimized 2-chlorophenol molecule relatively stable parallel (**b**), and vertical (**c**) adsorption on the pristine ZnO monolayer. The Fermi energy level is set to zero.

In order to better understand the electronic properties of 2-chlorophenol adsorption on 2D ZnO monolayers, the band structure and density of states were investigated. The band structures of pristine ZnO monolayer and adsorbed systems were calculated (as shown in Figure 3). In contrast to the pristine ZnO monolayer, both the conduction band and valence band of the adsorbed system move toward lower energy levels. When the 2-chlorophenol molecule adsorbs on the ZnO monolayer, a new flat band appears near the Fermi level (as shown in Figure 3b,c).

**Figure 4:**
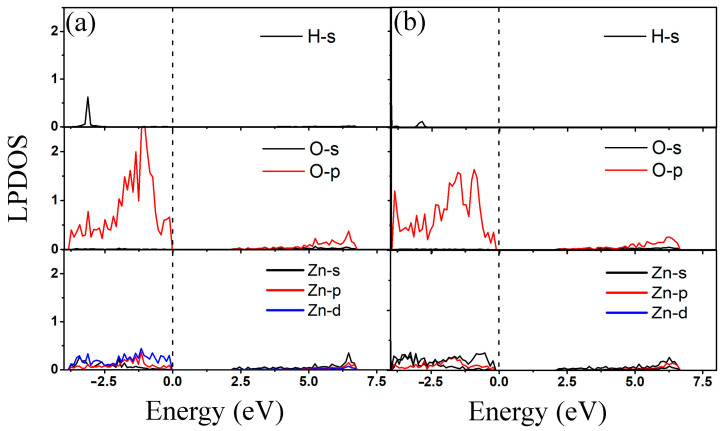
Local partial density of states of optimized 2-chlorophenol molecule relatively stable parallel (**a**) and vertical (**b**) adsorption on the pristine ZnO monolayer. The Fermi energy level is set to zero.

Local partial density of states (LPDOS) of 2-chlorophenol molecule adsorption on the pristine ZnO monolayer are calculated to obtain electron distribution properties. Figure 4 shows the LPDOS of 2-chlorophenol molecule adsorbed on the pristine ZnO monolayer based on the most stable adsorption configuration (parallel (a) and vertical (b) adsorption). From Figure 4, it can be seen that the electronic state near the Fermi level is mainly contributed by the 2p electron of the O atom of the 2-chlorophenol molecule. There is weak hybridization between the 2p electrons of oxygen atoms and the 3d electrons of Zn atoms near the Fermi level. Additionally, there is weak hybridization between H and O atoms at −2.0 eV. This once again indicates the weak adsorption between 2-chlorophenol molecules and the surface of ZnO.

### 3.2. Adsorption of 2-Chlorophenol on Al-Decorated ZnO Monolayers

We used an Al atom instead of a Zn atom to simulate the adsorption performance of 2-chlorophenol molecules on the surface of Al-modified 2D ZnO monolayer. Geometric parameters of the optimized 2-chlorophenol molecule adsorbed at different positions on a 2D Al-decorated ZnO monolayer are shown in Table 2. Compared with pure ZnO monolayer adsorbing 2-chlorophenol molecules, we also considered the adsorption configuration of 2-chlorophenol molecules on the top of Al. From Table 2, it can be seen that regardless of whether 2-chlorophenol molecules are adsorbed parallel or vertically on the surface of Al-modified ZnO monolayer, the corresponding adsorption energy at the top of Al is low. Therefore, Al modification of the ZnO surface can greatly improve the adsorption performance of 2-chlorophenol molecules. Using an Al-modified ZnO monolayer, 2-chlorophenol molecules transfer more charges to the surface of the ZnO monolayer, resulting in lower adsorption heights and energy. Previous studies have also reported that Al doping can improve the catalytic oxidation performance of ZnO for CO [43,44] and the adsorption performance of ZnO nanotubes toward methanol [47].

**Figure 5:**
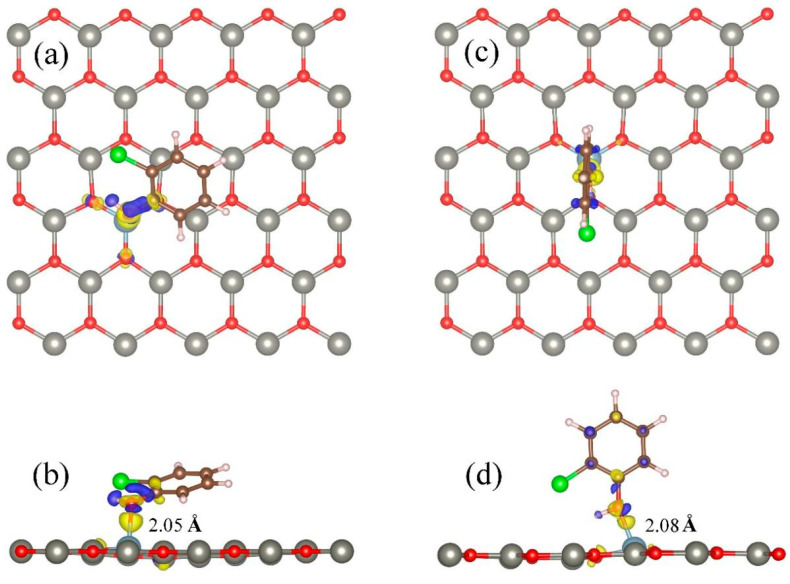
Charge density differences of optimized 2-chlorophenol molecule relatively stable parallel (**a**,**b**) and vertical (**c**,**d**) adsorption on Al-decorated ZnO monolayer. (**a**,**c**) TOP view (**b**,**d**) Side view. Yellow and blue correspond to charge accumulation and depletion, respectively. The isosurface value is 0.005 e/Å^3^. Red, gray, brown, green, white, and light blue spheres represent O, Zn, C, Cl, H, and Al atoms, respectively.

Figure 5 shows the charge density difference of stable adsorption (T_Al_) of optimized 2-chlorophenol molecules on Al-modified ZnO monolayers.

From Figure 5, it can be seen that compared to pristine ZnO monolayers, 2-chlorophenol molecules have more charge transfer with Al atoms. The bond length between the Al atom and the oxygen atom in the 2-chlorophenol molecule is only 2.05 (2.08) Ǻ, which is much smaller than the sum of their covalent radii. 2-Chlorophenol molecules form strong chemical bonds with surface Al atoms. This once again indicates that due to the doping effect of Al, the Al-modified ZnO monolayer has strong adsorption performance for 2-chlorophenol molecules. This feature can further promote the application of ZnO materials in the field of gas sensing.

**Figure 6:**
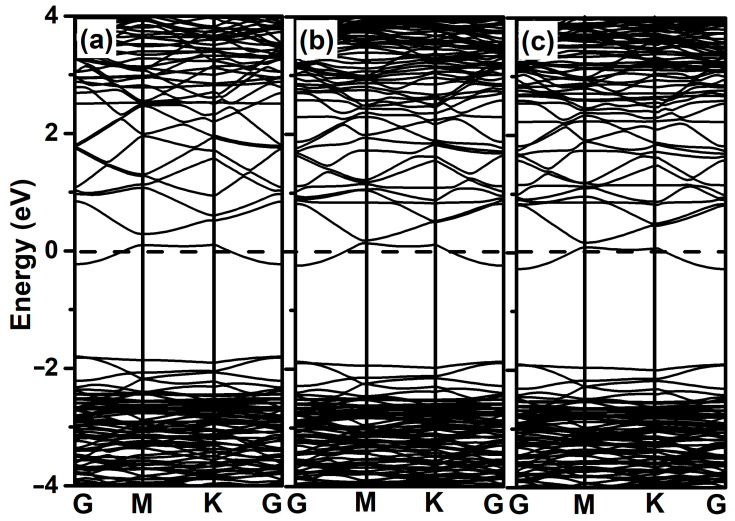
Band structures of Al-decorated ZnO monolayer (**a**), optimized 2-chlorophenol molecule relatively stable parallel (**b**), and vertical (**c**) adsorption on Al-decorated ZnO monolayer. The Fermi energy level is set to zero.

The energy band structures of Al-modified ZnO monolayer and 2-chlorophenol molecules adsorbed on it were calculated. As shown in Figure 6a, due to the modification of the Al atoms, new energy levels appear near the Fermi level of the ZnO monolayer. However, when 2-chlorophenol molecules adsorb onto the Al-modified ZnO monolayer, the band structure shows no significant difference except for a slight downward shift in the valence band, as shown in the Figure 6b,c.

**Figure 7:**
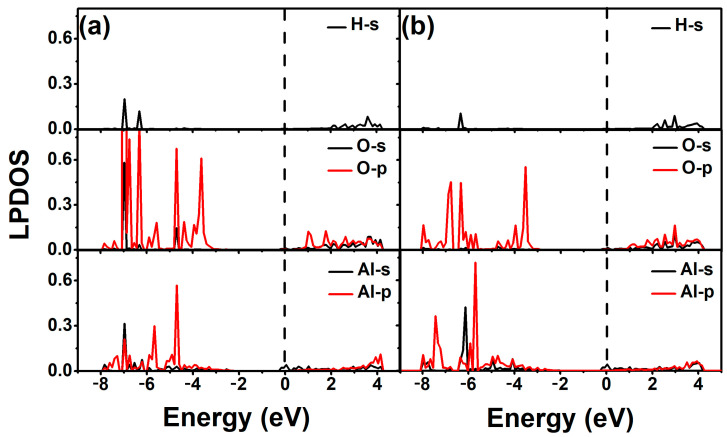
Local partial density of states of optimized 2-chlorophenol molecule relatively stable parallel (**a**) and vertical (**b**) adsorption on the Al-decorated ZnO monolayer. The Fermi energy level is set to zero.

In order to better understand the electronic properties of the binding between 2-chlorophenol molecules and Al-modified ZnO monolayers, we also calculated the local partial density of states of the optimized 2-chlorophenol molecule adsorbed on it. As shown in Figure 7, it is precisely due to the hybridization of 3p electrons of Al and 2p electrons of O that the adsorption performance of Al-modified ZnO monolayer for 2-chlorophenol molecules is enhanced. This feature also helps to understand the gas-sensing properties of Al-modified ZnO monolayers toward toxic gas molecules such as 2-chlorophenol molecules.

### 3.3. Dissociation of 2-Chlorophenol on Pristine and Al-Decorated ZnO Monolayers

**Figure 8:**
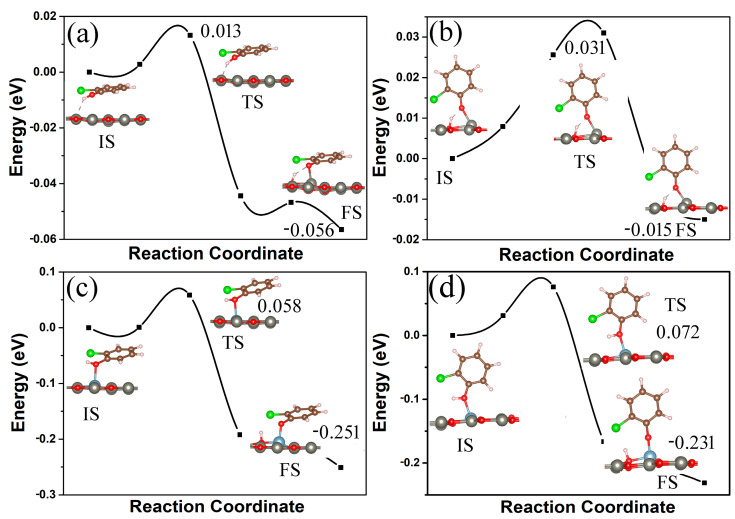
The dissociation pathways and energy profiles (in eV) for 2-chlorophenol on pristine (**a**,**b**) and Al-decorated ZnO (**c**,**d**) monolayers. Parallel (**a**,**c**) and vertical (**b**,**d**) adsorption. IS, TS, and FS represent the initial state, transition state, and final state, respectively. The initial state energy is set to zero eV. Yellow and blue correspond to charge accumulation and depletion, respectively. The isosurface value is 0.005 e/Å^3^. Red, gray, brown, green, white, and light blue spheres represent O, Zn, C, Cl, H, and Al atoms, respectively.

The dissociation of 2-chlorophenol to form chlorophenoxy radical is a key step in its degradation and conversion to PCDD/Fs. We investigated the dissociation of 2-chlorophenol molecules on clean ZnO and Al-modified ZnO monolayers using density functional theory (DFT) combined with the climbing image nudged elastic band method (CI-NEB) [53]. The dissociation pathways and energy profiles (in eV) for 2-chlorophenol on pristine and Al-decorated ZnO monolayers are shown in Figure 8. For a pristine ZnO monolayer, the energy required for the dissociation of parallel (vertical) adsorbed 2-chlorophenol molecules on its surface is only 0.013 (0.031) eV, as shown in Figure 8a,b. And the energy of the corresponding final state is only 0.056 (0.015) eV lower than that of the initial state. This indicates that the dissociation of 2-chlorophenol molecules on pure ZnO monolayer is extremely unstable, and the dissociated chlorophenoxy radical is prone to combine with H ions to form a 2-chlorophenol molecule again. However, as shown in Figure 8c,d, for the Al-modified ZnO monolayer, the final state energy of dissociated 2-chlorophenol molecules is much lower than the initial state energy by 0.251 (0.231) eV. This shows that the dissociated chlorophenoxy radicals can stably adsorb on the Al-modified ZnO monolayer. Moreover, the energy required for the dissociation of parallel (vertical) adsorbed 2-chlorophenol molecules on its surface is only 0.058 (0.072) eV. From this, it can be seen that due to the modification of Al metal atoms, a ZnO monolayer can serve as a potential material for 2-chlorophenol degradation.

## 4. Conclusions

In summary, the adsorption and dissociation characteristics of 2-chlorophenol molecules on both pristine and Al-modified ZnO monolayers were investigated through first-principles calculations. 2-chlorophenol molecules can be absorbed near the T_Zn_ site of the pristine ZnO monolayer with an exothermic process. By doping Al atoms, the adsorption capacity of the pristine ZnO monolayer for 2-chlorophenol can be greatly improved. The adsorption energy of 2-chlorophenol molecules on the Al-modified ZnO monolayer is −1.12 eV, which is much lower than the adsorption energy (−0.68 eV) on the pristine ZnO monolayer. Therefore, compared to ZnO monolayers, Al-atom-modified ZnO monolayers are more sensitive to 2-chlorophenol molecules. In addition, both the original ZnO and Al-modified ZnO monolayers exhibit lower potential barriers (0.013~0.072 eV) for the dissociation of H atoms in the hydroxyl group of 2-chlorophenol molecules. Our results provide insight into the adsorption and dissociation properties of ZnO monolayers, which could promote the further application of ZnO materials in the fields of catalysis and gas sensing.

## Figures and Tables

**Table 1:** Geometric parameters of the optimized 2-chlorophenol molecule adsorbed at different positions on a 2D ZnO monolayer. Adsorption energy (E_ad_), the adsorption height (h) between the O atom in 2-chlorophenol molecule and the 2D ZnO monolayer, and charge transfer (Q) between the 2-chlorophenol molecule and the 2D ZnO monolayer.

Orientation	Position	E_ad_ (eV)	h (Å)	Q (e)
parallel	T_Zn_	−0.68	2.56	0.063
	H	−0.61	2.91	0.055
	T_O_	−0.57	3.40	0.044
vertical	T_Zn_	−0.47	2.62	0.036
	H	−0.46	2.95	0.031
	T_O_	−0.45	3.44	0.025

**Table 2:** Geometric parameters of the optimized 2-chlorophenol molecule adsorbed at different positions on a 2D Al decorated ZnO monolayer. Adsorption energy (E_ad_), the adsorption height (h) between the O atom in 2-chlorophenol molecule and the 2D Al-decorated ZnO monolayer, and charge transfer (Q) between the 2-chlorophenol molecule and 2D Al-decorated ZnO monolayer.

Orientation	Position	E_ad_ (eV)	h (Å)	Q (e)
parallel	T_Al_	−1.12	2.05	0.153
	T_Zn_	−0.70	2.55	0.073
	H	−0.62	2.90	0.064
	T_O_	−0.58	3.39	0.050
vertical	T_Al_	−0.97	2.08	0.103
	T_Zn_	−0.48	2.60	0.043
	H	−0.47	2.92	0.041
	T_O_	−0.46	3.42	0.030

## Data Availability

The original contributions presented in the study are included in the article, further inquiries can be directed to the corresponding author.
